# Mesenchymal Stem Cells Promote Liver Regeneration and Prolong Survival in Small-For-Size Liver Grafts: Involvement of C-Jun N-Terminal Kinase, Cyclin D1, and NF-κB

**DOI:** 10.1371/journal.pone.0112532

**Published:** 2014-12-05

**Authors:** Weijie Wang, Zhiyong Du, Jiqi Yan, Di Ma, Minmin Shi, Mingjun Zhang, Chenghong Peng, Hongwei Li

**Affiliations:** 1 Department of Surgery, Ruijin Hospital, Shanghai Jiaotong University School of Medicine, Shanghai, China; 2 Department of Surgery, First Affiliated Hospital of Zhengzhou University, Zhengzhou University School of Medicine, Zhengzhou, Henan Province, China; 3 Department of Hepatobiliary Surgery, Central Hospital of Wuhan, Wuhan, Hubei Province, China; Indiana University School of Medicine, United States of America

## Abstract

**Background:**

The therapeutic potential of mesenchymal stem cells (MSCs) has been highlighted recently for treatment of acute or chronic liver injury, by possibly differentiating into hepatocyte-like cells, reducing inflammation, and enhancing tissue repair. Despite recent progress, exact mechanisms of action are not clearly elucidated. In this study, we attempted to explore whether and how MSCs protected hepatocytes and stimulated allograft regeneration in small-for-size liver transplantation (SFSLT).

**Methods:**

SFSLT model was established with a 30% partial liver transplantation (30PLT) in rats. The differentiation potential and characteristics of bone marrow derived MSCs were explored in vitro. MSCs were infused transvenously immediately after graft implantation in therapy group. Expressions of apoptosis-, inflammatory-, anti-inflammatory-, and growth factor-related genes were measured by RT-PCR, activities of transcription factors AP-1 and NF-κB were analyzed by EMSA, and proliferative responses of the hepatic graft were evaluated by immunohistochemistry and western blot.

**Results:**

MSCs were successfully induced into hepatocyte-like cells, osteoblasts and adipocytes in vitro. MSCs therapy could not only alleviate ischemia reperfusion injury and acute inflammation to promote liver regeneration, but also profoundly improve one week survival rate. It markedly up-regulated the mRNA expressions of HGF, Bcl-2, Bcl-XL, IL-6, IL-10, IP-10, and CXCR2, however, down-regulated TNF-α. Increased activities of AP-1 and NF-κB, as well as elevated expressions of p-c-Jun, cyclin D1, and proliferating cell nuclear antigen (PCNA), were also found in MSCs therapy group.

**Conclusion:**

These data suggest that MSCs therapy promotes hepatocyte proliferation and prolongs survival in SFSLT by reducing ischemia reperfusion injury and acute inflammation, and sustaining early increased expressions of c-Jun N-terminal Kinase, Cyclin D1, and NF-κB.

## Introduction

Ever since the first clinical successful attempt of cadaveric split liver transplantation and living donor liver transplantation in the late 1980′s, those approaches have been considered the optimal procedure for end-stage liver disease due to the increasing shortage of cadaveric donors [1], [2]. After partial liver transplantation, rapid liver regeneration is required to ensure sufficient liver function and prevent small-for-size syndrome [3]. Nevertheless, transient portal hypertension, ischemia reperfusion injury and subsequent severe inflammatory responses at the early phase after cadaveric split liver transplantation or living donor liver transplantation may delay hepatocyte proliferation and even result in small-for-size graft failure [4]–[6]. Therefore, effective therapeutic strategies aimed at reducing ischemia reperfusion injury and acute inflammatory responses to promote the regeneration of hepatocytes would be of great benefit.

Mesenchymal stem cells (MSCs), also called multipotent mesenchymal stromal cells, are on the brink of being used clinically in different areas of therapeutic application, including organ transplantation [7]–[9]. They are defined as plate-adherent, fibroblast-like cells possessing self-renewal ability with the capacity to differentiate into multiple mesenchymal cell lineages such as hepatocyte-like cells, osteoblasts, chondrocytes, and adipocytes [10], [11]. It is also well known of their ability to naturally support hematopoiesis by secreting a number of trophic molecules, including soluble extracellular matrix glycoproteins, cytokines, and growth factors [3], [7]. Various studies have demonstrated the therapeutic potential of MSCs in different liver disease models [7], [12], [13], such as liver resection [14], [15], fulminant hepatic failure [16], [17], liver fibrosis [10], [11], [18]–[20], and liver transplantation [9], [21], [22]. Its multilineage differentiation potential and anti-inflammatory properties, as well as producing trophic factors to provide paracrine support for hepatocyte proliferation, angiogenesis, tissue repair, and immunomodulation, have been proposed to play a key point in rescuing liver injury [9], [23]. Moreover, their easy accessibility and strong in vitro expansion ability make them an ideal cell source for autologous stem-cell-based replacement therapies [7], [24].

To date, however, a few investigations have been done on the potential use of MSCs to improve the outcome of small for size liver transplantation (SFSLT). The therapeutic mechanisms of MSCs are also not clearly elucidated. Whether MSCs contribute to liver regeneration by trans-differentiation into liver cells or by paracrine effects of their trophic factors has been ongoing discussions [3]. Both Zhong Z and our previous study have already confirmed that defective liver regeneration in small grafts was associated with an inhibition of the c-Jun N-terminal kinase (JNK/c-Jun) and cyclin D1 (CyD1) pathways and compromised energy production [4], [5]. We also found that MSCs-conditioned medium could reduce liver injury and enhance regeneration in 50% reduced-size rat liver transplantation [25]. Therefore, in this study we sought to determine whether and how MSCs therapy promoted liver regeneration and subsequently prolonged the rat survival in 30% SFSLT.

## Materials and Methods

### Ethics statement

All animal care and experimental procedures complied with the guidelines for the Care and Use of Laboratory Animals, formulated by the Ministry of Science and Technology of the People's Republic of China, and were approved by the Ethical Committee on Animal Experiments at Ruijin Hospital (protocol approval number SYXK 2011–0113).

### Isolation and culturing of MSCs

Three-week-old male inbred Sprague Dawley rats were euthanized, and bone marrow derived MSCs were harvested by flushing the femurs and tibias with sterile PBS and spinning down the cellular content. After resuspension in α-MEM, cells were filtered through 70 µm mesh and plated in 75 cm^2^ primary culture flasks with α-MEM/Ham's F-12 (Sigma, USA) and 10% fetal bovine serum (FBS). Nonadherent cells were removed after 72 h, and adherent cells were passed at low density into new flasks. MSCs had a typical spindle-shaped appearance, and the 3rd-passage MSCs were used in the experiments.

### Identification of MSCs by flow cytometry

At least 2×10^5^ MSCs were harvested and resuspended in 0.1 ml PBS containing 1% bovine serum albumin (Sigma, USA). The cell suspension was incubated with 0.2 µg fluorescein isothiocyanate- or phycoerythrin-conjugated primary antibody (1∶100 dilution), mouse monoclonal anti-rat CD34 (Santa Cruz Biotechnology, USA), anti-CD90, anti-CD29 (BioLegend, USA) and anti-CD45 (Catag Laboratories, USA) for 40 minutes at 4°C. The mouse IgG1 kappa antibody (Catag Laboratories, USA) was used as an isotype control. MSC surface markers were analyzed by fluorescence-activated cell sorter (BD Calibur, USA).

### Hepatogenic differentiation of MSCs

Hepatogenic differentiation was performed with reference to previous research [26], [27]. Briefly, the cultured cells were harvested from the culture bottles with 0.25 g/L trypsin. Then the 3rd-passage cells were seeded in 6-well cell culture plates. When the cells grew to 80% confluence, the control group was continuously cultured in α-MEM (10% FBS). The hepatocyte differentiation group was cultured in α-MEM (10% FBS; 10^−7^ µmol/L dexamethasone, DXM; 1× Insulin-Transferrin-Selenium, ITS; 20 ng/mL hepatocyte growth factor, HGF; 20 ng/mL fibroblast growth factor-4, FGF-4; 20 ng/mL epidermal growth factor, EGF). In each well, 2 mL of medium was added and changed every 4 days. To determine the cell phenotype, the cultured cells were stained by anti-alpha-fetoprotein (AFP) and anti-albumin (ALB) protein monoclonal antibodies (Santa Cruz Biotechnology, USA) according to the manufacturer's protocol. The presence of insoluble stored glycogen content in the trans-differentiated cells was assessed by periodic acid Schiff's (PAS) staining, for which the cells at days 28 and 35 were fixed with 4% paraformaldehyde for 30 minutes. It was then oxidized with 1% periodic acid for 5 min, washed and incubated with Schiff's reagent for 15 min. The cells were washed and stained with hematoxylin, and then observed under light microscope. Uninduced MSCs were used as negative control.

### Osteogenic differentiation of MSCs

MSCs were cultured in 6-well plates at 3100 cells/cm^2^ with α-MEM (10% FBS). At subconfluence, the medium was changed with an osteoblast medium (Cyagen Biosciences, USA). All the differentiation process was in strict accordance with the kit instructions (Cyagen Biosciences, USA). 21 days later, the cells were paraformaldehyde-fixed and were tested for calcium with Alizarin red.

### Adipogenic differentiation of MSCs

MSCs were cultured in 6-well plates at 21000 cells/cm^2^ in α-MEM (10% FBS). The medium was changed with an adipocyte medium (Cyagen Biosciences, USA) after 24 hours. All the differentiation process was in strict accordance with the kit instructions (Cyagen Biosciences, USA). 28 days later, the cells were paraformaldehyde-fixed, and lipid vacuoles were tested with Sudan red.

### Surgical procedure and experimental design

Male inbred Sprague Dawley rats (220–240 g) were used as both donors and recipients. Rats were housed in a standard animal laboratory with free activity and access to water and chow. They were kept under constant environmental conditions with a 12-hour light-dark cycle. The rats were fasted for 12 hours before surgery. Operations were performed under clean conditions.

SFSLT model was established with a 30% partial liver transplantation (30PLT) in rats. They were anesthetized by intraperitoneal injection of 1% pentobarbital sodium 40 mg/kg. Non-arterialized orthotopic liver transplantation was performed as described previously [28]. The lobe ligation technique was used to reduce the graft size on the back table. In detail, the reduced graft used for 30% partial liver transplantation was composed of the superior portion and the inferior portion of the right lobe, the anterior caudate lobe, and the posterior caudate lobe. The average cold ischemia time was about 60 minutes, and the average anhepatic phase was about 20 minutes.

Rats undergoing 30% partial liver transplantation were randomly divided into two groups: 30PLT+PBS (Phosphate buffered saline; n = 25) and 30PLT+MSCs (n = 25). Both groups were further randomly divided into 5 subsections including rats with 30% PLT 1 h, 6 h, 24 h, 72 h, and 7days. Each subgroup contained 5 rats. As systemic infusion of MSCs via inferior vena cava could not only achieve the same effects as peripheral veins route (the vena caudalis or penile vein) with a low probability of pulmonary emboli, but also avoid the defects of portal vein route, which is easy to cause bleeding, obstruct hepatic sinusoid and aggravate liver necrosis. Immediately after hepatic graft reperfusion, 2.4×10^6^ MSCs in a volume of 0.5 ml PBS was slowly transfused via inferior vena cava of each recipient rat with 28-gauge needle in the 30PLT+MSCs group. Such a dosage of MSCs was proved effective and with little complications in our preliminary experiments. Only PBS-treated 30PLT rats served as controls. At the corresponding time points, animals were sacrificed under anesthesia by exsanguination from abdominal aorta, as described in our previous study [29].

### Graft weight, histology and immunohistochemistry

Graft weight before and after liver transplantation was recorded in both groups, and the increase ratio was calculated. Liver biopsy specimens were taken at 1 h, 6 h, 24 h, 3 days and 7 days after reperfusion. Formalin-fixed and paraffin-embedded liver tissue was used for evaluation of liver pathological changes after Hematoxylin-Eosin (HE) staining. Liver biopsies were also examined under a transmission electron microscope. The liver biopsies were immediately cut into 1-mm cubes and fixed in 2.5% glutaraldehyde at 4–8°C for electron microscopy section.

In addition, paraffin sections of the liver biopsies were immunochemically stained for PCNA and CyD1 using the Dako EnVision system (Dako, Denmark). In brief, after deparaffinization, endogenous peroxidase activity was quenched by immersing the sections for 30 min in absolute methanol containing 0.3% H_2_O_2_. The sections were processed to unmask the antigens by conventional microwave oven heating in 10 M citric acid buffer (pH 6.0) for 12 min. The sections were then treated with 30% normal goat serum for 30 min to reduce the background staining, followed by treatment with appropriate primary antibodies PCNA (Epitomics, USA) and CyD1 (Epitomics, USA) at 4°C overnight. After washing, the sections were incubated with EnVision for 30 min at room temperature and then visualized with a chromogenic substrate solution for 2 min. The slides were examined under a light microscope. Morphometry was performed from three random fields of every slide using the Image-Pro plus software (Media Cybernetics Inc, USA). For each microscopic field, the positive area was calculated automatically by the software, and this positive area was in turn divided by the total area of the microscopic field. Morphometric results were then expressed as volume fractions (percentage of specific positive area in relation to the total counted area).

### The detection of intrahepatic engraftment of MSCs

GFP-labelled MSCs were used to detect the hepatic engraftment of transplanted MSCs on the third (n = 5) and seventh (n = 5) day after infusion. Expression vector pEGFP-N3 (Clontech, USA) was transfected into MSCs using lipofectamine transfection reagent (Invitrogen, USA) according to manufacturer's recommended procedures. The transfected cells were cultured in α-MEM medium containing 400 µg/ml of geneticin G418 (Merck, Germany). Selection of GFP-positive clones was conducted by fluorescent microscope to isolate green fluorescence cells for further expansion. Stability of GFP-labelled MSCs was obtained after repeated clonal passages for 5 weeks. The liver tissue sections were washed in PBS and then incubated with 4′,6-diamidino-2-phenylindole (DAPI). Finally, images were captured by fluorescent microscope (Nikon, Japan).

### RNA isolation and quantitative real-time PCR

Samples of liver tissues were taken from the hepatic grafts at 1, 6, 24 and 72 h after reperfusion. Total RNA was isolated from about 30 mg of liver tissue with Trizol Reagent (Invitrogen, USA) according to the manufacturer's instructions. Then the reverse transcription was done using a Reverse Transcription Kit (TOYOBO, Japan). Quantitative real-time PCR was carried out in triplicate using SYBR Green Master Mix (TOYOBO, Japan) on a 7900HT Fast Real-Time PCR System (Applied Biosystems, USA). The rat nucleotide sequences and accession numbers of primers were summarized in Table 1. Data were normalized to the housekeeping gene glycerinaldehyd-3-phosphatdehydrogenase (GAPDH).

**Table 1:** Nucleotide sequences of RT-PCR primers.

Target genes	Primer pairs (Forward/Reverse)	Accession number
Bcl-2	CTGGCATCTTCTCCTTCC	NM 016993.1
	GAGTTCCTCCACCACCGT	
Bcl-XL	GCTGGTGGTTGACTTTCTC	NM 001033672.1
	AGTTTCTTCTGGGGCTTC	
CXCR2	CATCCTGCCTCAGACCTA	NM 017183.1
	AAGCCAAGAATCTCAGTAGC	
HGF	TCATTGGTAAAGGAGGCA	NM 017017.2
	GTCACAGACTTCGTAGCGTA	
IL-6	TCCGCAAGAGACTTCCAGCCAGT	NM 012589.2
	AGCCTCCGACTTGTGAAGTGGT	
IL-10	AGCCAGACCCACATGCTCCGA	NM 012854.2
	ACAGGGGAGAAATCGATGACAGCGT	
IP-10	CTGCACCTGCATCGACTTCC	NM 139089.1
	TTCTTTGGCTCACCGCTTTC	
TNF-α	CCACGCTCTTCTGTCTACTG	NM 012675.3
	CTACGGGCTTGTCACTCG	
GAPDH	TACAAGGAGTAAGAAACCGTGGAC	NM 017008.4
	GTTATTATGGGGTCTGGGATGG	

The results were expressed as the number of cycles (CT value) at which the fluorescence signal exceeded a defined threshold. The difference in CT values of the target cDNA and GAPDH was expressed as △CT values. Therefore, lower △CT values denoted higher mRNA levels. The 2^−_△_^CT^^ method was used for quantification of the results.

### Electrophoretic mobility shift assay (EMSA)

Liver tissues were snap-frozen at 1, 6 and 24 h after implantation and stored at −80°C. The tissues were homogenized and nuclear protein was extracted with a NE-PER Nuclear and Cytoplasmic kit (Pierce, USA). The levels of activator protein 1 (AP-1) and nuclear factor (NF)-κB were then measured by EMSA, as described elsewhere [30]. Detailed information of biotin-conjugated AP-1 and NF-κB consensus oligonucleotides is presented in Table 2. The signal intensities were determined by enhanced chemiluminescence (ECL) reagents, and acquired with a Molecular Imager ChemiDox XRS+ Imaging System with Quantity One Image software (Bio-Rad, USA). ImageJ software was used to quantify the gray value.

**Table 2:** Oligonucleotide sequence of transcription factor detected by EMSA.

	Oligonucleotide sequence
AP-1	5′-CGC TTG ATG ACT CAG CCG GAA-3′
	3′-GCG AAC TAC TGA GTC GGC CTT-5′
NF**-**κB	5′-AGT TGA GGG GAC TTT CCC AGG C-3′
	3′-TCA ACT CCC CTG AAA GGG TCC G-5′

### Western blot analysis

Samples of liver tissues were taken from the hepatic grafts at 6, 24 and 72 h and 7 days after reperfusion in recipients. Parts of them were put in ice-cold Tissue Protein Extraction Reagent (Pierce, USA) containing a mixture of proteinase inhibitors cocktails (Sigma, USA) and phosphatase inhibitors (Roche, Switzerland) to be homogenated by the tissue homogenizer. Samples were then centrifuged for 30 minutes at 10000 g. The supernatant was collected, and the protein concentration was measured using the BCA Protein Assay Kit (Pierce, USA). Proteins (80∼120 µg) were separated by SDS-PAGE and subsequently transferred to polyvinylidenefluoride (PVDF) membranes. The membranes were blocked with 5% bovine serum albumin (BSA) in incubation buffer for 2 h at room temperature. Afterwards, they were incubated with antibodies against p-c-Jun (Epitomics, USA) and CyD1 and GAPDH (Abcam, USA) at 4°C overnight and then with the peroxidase-conjugated secondary antibody at room temperature for 2 h. Finally, the signal was detected by ECL. Loading accuracy was evaluated by membrane rehybridization with anti-GAPDH antibodies. Bands were acquired with a Molecular Imager ChemiDox XRS+ Imaging System with Quantity One Image software and quantified with ImageJ software.

### Survival study

Another 24 rats were randomly divided into two groups (30PLT+PBS, n = 12; 30PLT+MSCs, n = 12) by a randomisation list and used for survival rate study. In order to minimize experimental error and suffering of the rats, we set humane endpoints to determine when to kill the rats. The humane endpoints included that spiritual malaise, limbs collapse,intermittent stimulation 3 times to the rat without significant response within half an hour, the temperature was significantly lower than the active rats and it did not increase or contrarily decrease within a few hours, and breathing significantly sped up or slowed down obviously. We monitored the condition of the animals every 6 h and strictly abode by the humane endpoints to determine when to sacrifice the rats. Rats which had lived for more than 7 days after transplantation were considered survivors.

### Statistical analysis

Comparison between the two groups was by Mann Whitney U Test using the GraphPad statistics software (GraphPad Software Inc, USA). All results indicated means ± standard deviation (SD). Both the columnar diagrams and survival curves were made by GraphPad Software. Results were considered statistically significant with a p-value <0.05.

## Results

### Characteristics of MSCs

**Figure 1:**
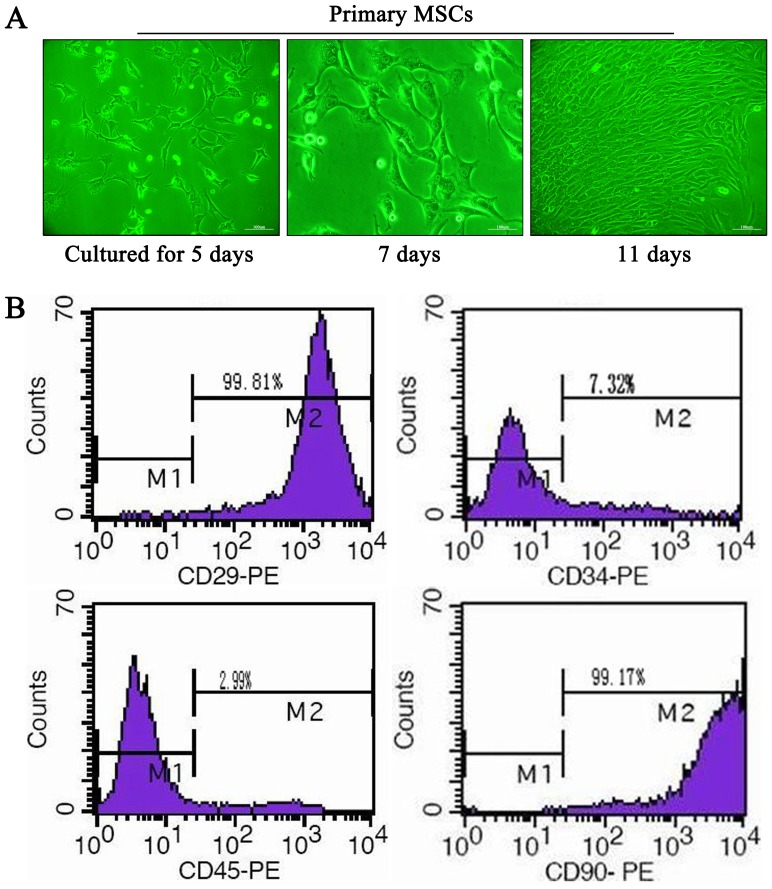
Mesenchymal stem cell (MSC) culture and identification. (A) The morphology of MSCs cultured at 5th, 7th, and 11st day (magnification ×100); (B) Fluorescence-activated cell sorting analysis of rat MSCs. Numbers in the panels represent percentage of the cells expressing each marker.

MSCs were generated by standard procedures and grown for at least three passages in culture. Contaminating hemotopoietic cells were depleted during passaging, and MSCs were morphologically defined by a fibroblast-like appearance and their identity was confirmed by flow cytometry (Fig 1). Over 95% of the isolated MSCs expressed CD29 and CD90, but not CD34 or CD45. These results are in accordance with well-established markers of bone marrow-derived MSCs.

### MSCs differentiate into hepatocyte-like cells, osteoblasts and adipocytes in vitro

**Figure 2:**
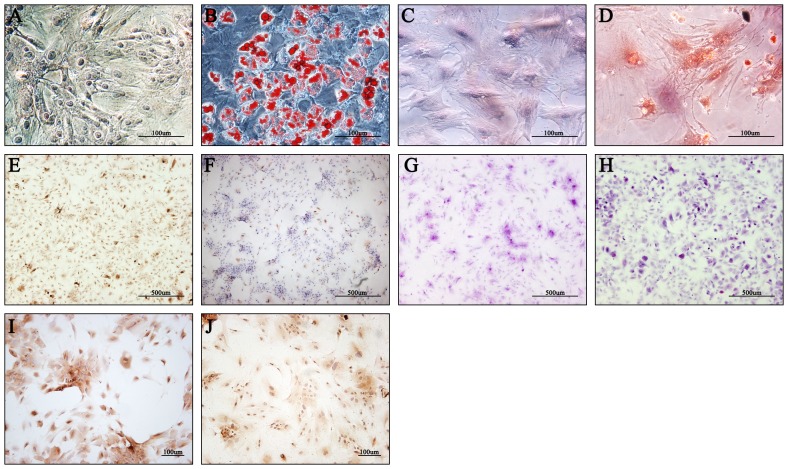
Mesenchymal stem cells (MSCs) were successfully induced into hepatocyte-like cells, osteoblasts and adipocytes in vitro. Sudan red staining of (A) MSCs (magnification ×200) as control, and (B) adipocytes that were differentiated from MSCs at 28th day (magnification ×200). Alizarin red staining of (C) MSCs (magnification ×200) as control, and (D) osteoblasts that were differentiated from MSCs at 21st day (magnification ×200). Immunohistochemical staining for AFP of hepatocyte-like cells induced for (E) 14 and (F) 21 days respectively (magnification ×40). Periodic acid-Schiff (PAS) staining for glycogen storage of hepatocyte-like cells induced for (G) 28 and (H) 35 days respectively (magnification ×40). Immunohistochemical staining for ALB of hepatocyte-like cells induced for (I) 21 and (J) 28 days respectively (magnification ×100).

As for the differentiation into osteoblasts and adipocytes, MSCs were cultured in the corresponding differentiation medium. MSCs seeded in the ordinary medium were as control (Fig 2AC). After exposure for 21 days and 28 days respectively, osteoblasts (Fig 2D) and adipocytes (Fig 2B) were verified by Alizarin and Sudan red staining of intracellular calcium and fat deposits respectively. The results proved that the cells were undifferentiated and had stem cell characteristics.

When cultured MSCs reached 80% confluence, they were seeded in hepatocyte differentiation medium. After 6∼8 days induction, part of MSCs began to display changes in cellular morphology including shrinkage of the cytoplasm and diameter, and became in round cells. About 18 days in culture, all MSCs were nearly transformed into homogeneous hepatocyte-like cells. The control MSCs did not exhibit such changes in morphology with ordinary culture medium. Furthermore, MSCs stained positive for AFP and ALB hepatocyte markers 14 days after induction. However, expression of AFP on 21st day (Fig 2F) was much less than that on 14th day (Fig 2E) in trans-differentiated hepatocyte-like cells. On the contrary, expression of ALB on 28th day (Fig 2J) was more than that on 21st day (Fig 2I), and gradually increased with extended exposure. Consistently, glycogen content also evidently increased on 35th day (Fig 2H) compared with that on 21st day (Fig 2G). These results indicated that MSCs differentiation was more and more close to mature hepatocytes following exposure.

### MSCs therapy improves intra-graft morphological structure and shows rare intrahepatic engraftment

**Figure 3:**
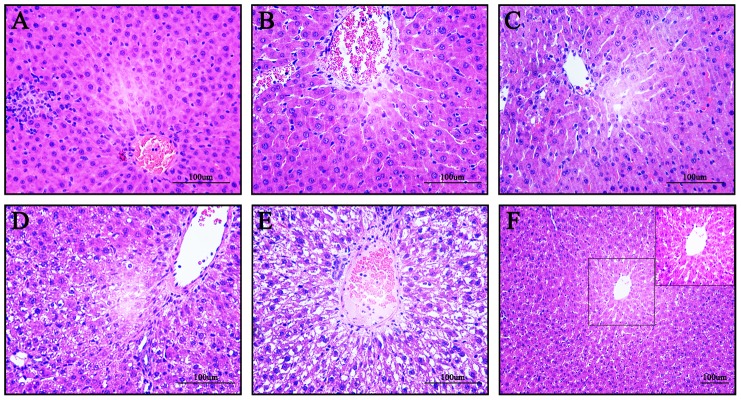
Intra-graft histopathological changes were improved after MSCs transplantation. Hematoxylin and Eosin (HE) Staining of livers after 30PLT+PBS (A) 1 h, (B) 6 h, (C) 1 day, (D) 3 days, and (E) 7 days. (F) HE Staining of livers after 30PLT+MSCs 7 days.

Within 24 h after liver transplantation, little graft weight increase could be observed in either group. 3 days later, the increase ratios of graft weight in the 30PLT+PBS and 30PLT+MSCs groups were 19.9±3.6% and 74.5±12.4% respectively, and became 99.8±7.4% and 127.2±10.1% 7 days later. These results implied that MSCs therapy could early and rapidly promote liver regeneration. Unsurprisingly, we demonstrated a serious histological damage in hepatic grafts of the 30PLT+PBS group. HE-staining showed a dynamic evolving process of tissue injury at 1 h, 6 h, 1 day, 3 days and 7 days after SFSLT (Fig 3). Obviously, the intrahepatic structure disorder and vacuoles degeneration gradually increased after surgery within 7 days. However, MSCs therapy showed a prominent protective effect and even reversed the intrahepatic acute injury response in graft.

**Figure 4:**
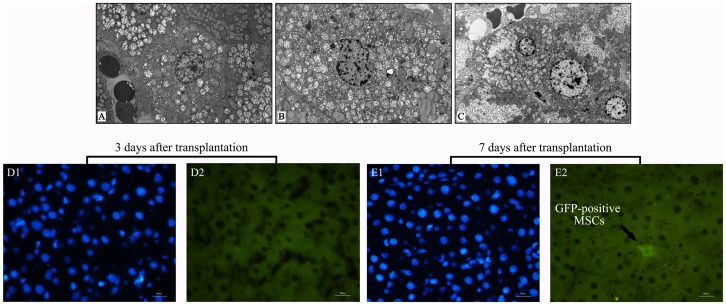
Electron microscopic examination of hepatic grafts at 24 h after reperfusion. In the 30PLT+PBS group (magnification ×4200), (A) the integrity of endothelial cells was disrupted, and the mitochondria exhibited diffuse swelling with vacuoles. (B) Shape of hepatocyte nuclei became irregular and even appeared pyknotic. The mitochondrial swelling with cristae rupture was also apparently visible. (C) In the 30PLT+MSCs group (magnification ×3400), both hepatocytes and sinusoid cells showed relatively normal microstructure. (D) 3 and (E) 7 days after transplantation of GFP-labelled MSCs. Sections were counterstained with DAPI (D1 and E1) and positive signal was shown by arrow (D2 and E2).

Consistently, our electron microscopy further confirmed the therapeutic effect of MSCs. Mitochondrial swelling of hepatocytes could be observed at 1 h after reperfusion and deteriorated 6 h later accompanied with hepatic vacuolar degeneration, sinusoidal congestion, as well as irregular gaps among sinusoidal lining cells in the control group. 24 h later, hepatocytes gradually exhibited cell shrinkage and karyopyknosis. Cell linkage was destroyed. The integrity of endothelial cells was disrupted and even apoptotic cells could be observed inside the sinusoids (Fig 4AB). However, MSCs therapy showed a protection for both the hepatocytes and sinusoidal lining cells, which basically sustained normal structure with a slight injury at different time points within 24 h after liver transplantation. In addition, the chromatin in the nucleus also displayed normal structure, and mitochondria were elliptical with well-visualized cristae. The endoplasmic reticulum was intact, and abundant microvilli in the space of Disse were visible in the MSCs therapy grafts (Fig 4C).

As for the detection of hepatic engraftment of transplanted MSCs, we searched for GFP-labelled MSCs on the third and seventh day after infusion (Fig 4DE). However, it was very difficult to find GFP-labelled MSCs in grafts, since we could only occasionally see a few GFP-positive MSCs on individual slices.

### MSCs therapy up-regulates the genes of HGF, Bcl-2, Bcl-XL, IL-10, IL-6, IP-10 and CXCR2, but down-regulates TNF-α

**Figure 5:**
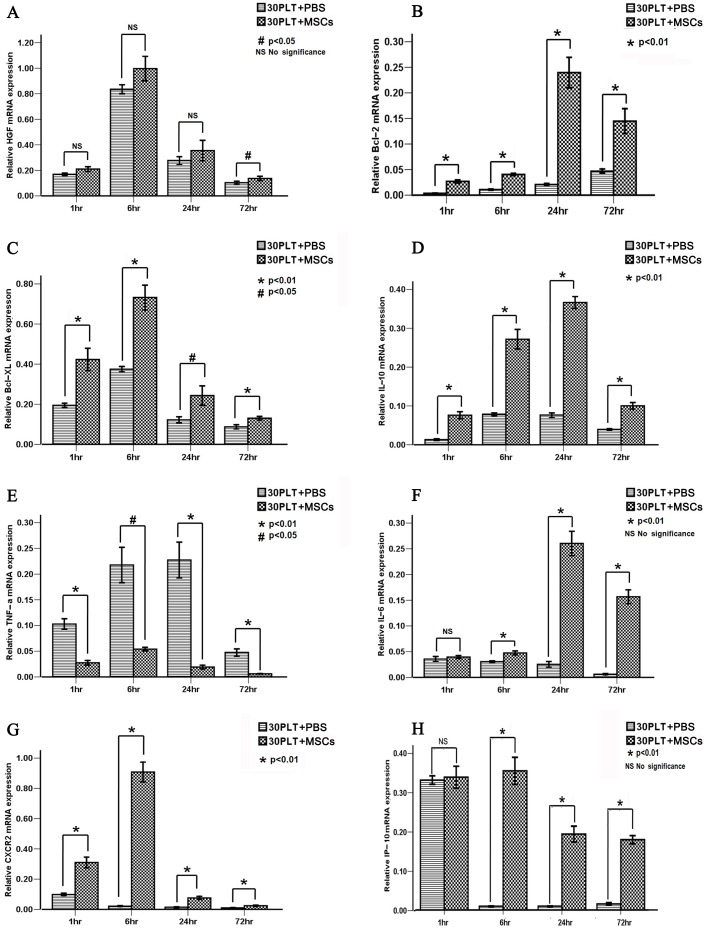
MSCs therapy up-regulated the genes of HGF, Bcl-2, Bcl-XL, IL-10, IL-6, IP-10 and CXCR2, but down-regulated TNF-α. Apoptosis-, inflammatory-, anti-inflammatory- and growth factor-related genes were screened at 1, 6, 24 and 72 h after reperfusion by real-time quantitative reverse transcription polymerase chain reaction (RT–PCR), including (A) HGF, (B) Bcl-2, (C) Bcl-XL, (D) IL-10, (E) TNF-α, (F) IL-6, (G) CXCR2 and (F) IP-10. (Mean ± SD; ^#^p<0.05; *p<0.01; NS- nonsignificant.)

To elucidate the underlying mechanisms of MSCs in promoting liver regeneration of SFSLT, the expression of several growth factor-, anti-apoptosis-, anti-inflammatory-, and inflammatory-related genes were quantified by RT-PCR (Fig 5).

HGF is secreted by mesenchymal cells and acts as a multi-functional cytokine. Its ability to stimulate mitogenesis, cell motility, and matrix invasion gives it a central role in angiogenesis and tissue regeneration. In this study, we found that the mRNA level of HGF in the MSCs therapy group showed an increased tendency at 1, 6 and 24 h after reperfusion, and displayed a significant up-regulation at 72 h compared with the controls. Moreover, the anti-apoptosis genes Bcl-2 and Bcl-XL were also significantly up-regulated postoperatively in the 30PLT+MSCs group versus the 30PLT+PBS group.

As for the anti-inflammatory cytokine interleukin (IL)-10, the mRNA level in the 30PLT+PBS group remained at a low level after reperfusion, however, MSCs therapy markedly increased its expression in the 30PLT+MSCs group. IL-6, which acts as both a pro-inflammatory cytokine and an anti-inflammatory myokine, showed a great benefit in reducing oxidative injury and necrosis after extreme liver resection [31] [30]. It continuously maintained low levels in the 30PLT+PBS group after reperfusion, however, MSCs therapy significantly up-regulated its transcription at 24 h after reperfusion and even at a comparatively high level at 72 h. On the other hand, the peak expression of pro-inflammatory TNF-α was observed at 6 h after reperfusion in the two groups, but MSCs therapy evidently reduced its expression at different time points compared with the controls.

Furthermore, chemokine IFN-γ inducible protein-10 (IP-10) and CXCR2 are usually present during various forms of acute and chronic liver injury, and modulate tissue inflammation by recruiting neutrophils or T cells from the spleen or bone marrow [32]. Encouragingly, a hepatoregenerative effect of them has been verified in acute liver injury [33]. In this study, we found a relative higher mRNA level of IP-10 in both groups at 1 h after reperfusion, but its expression dropped to low levels from 6 h in 30PLT+PBS group. Contrarily, MSCs therapy obviously increased its expression at 6, 24 and 72 h after transplantation in 30PLT+MSCs group. For the CXCR2 transcription, it showed a continuous high expression in the MSCs therapy group compared with the controls.

### MSCs therapy promotes the expressions of PCNA and CyD1 in graft

**Figure 6:**
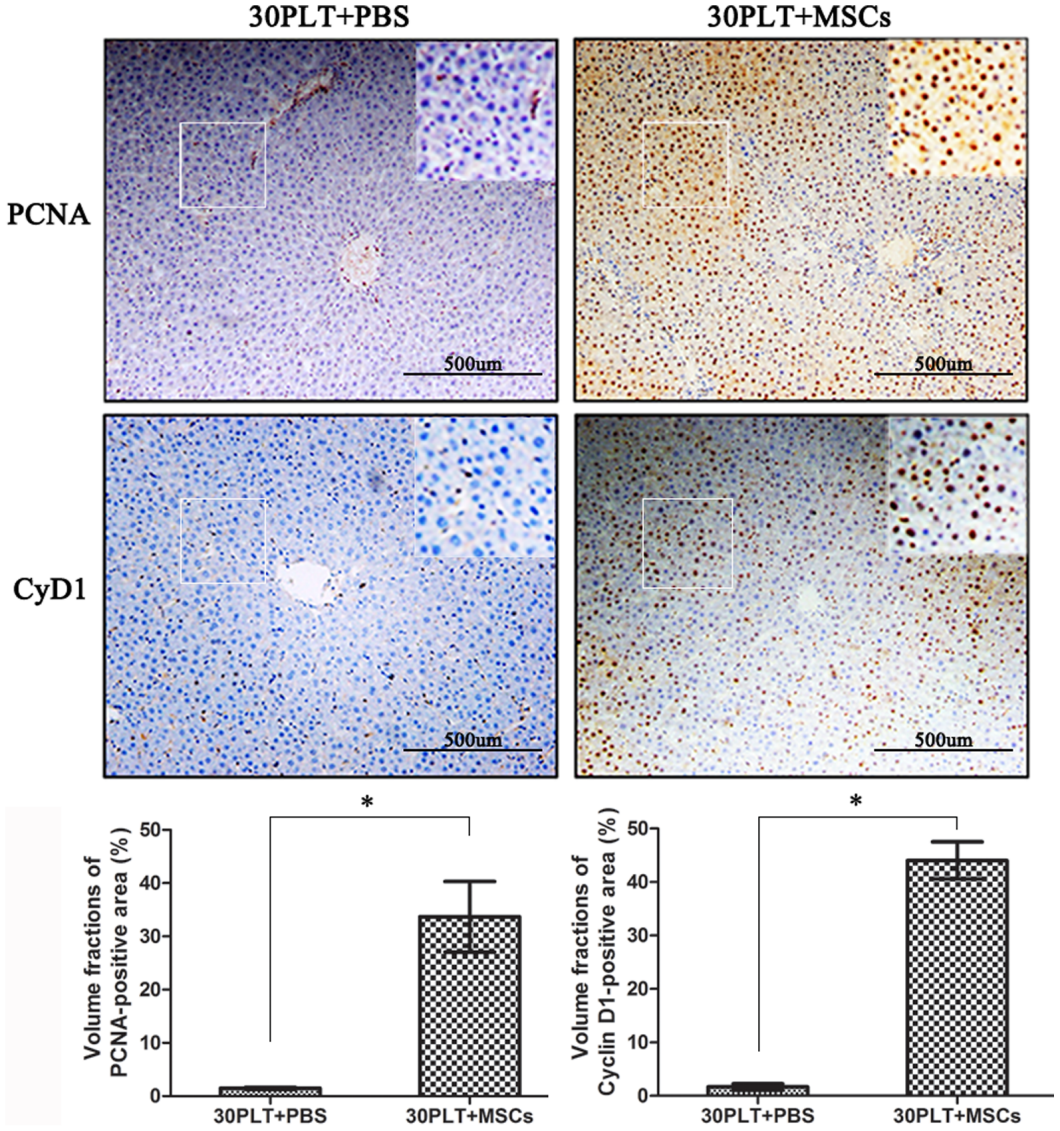
MSCs therapy sustained the active proliferative responses in the 30PLT+MSCs group. Immunochemical staining for PCNA and cyclin D1 at 72 h after reperfusion (magnification ×100). Compared with the 30PLT+PBS group, MSCs therapy markedly promoted the expressions of PCNA and CyD1 in graft. (Mean ± SD; *p<0.01.)

The hepatic proliferative responses were evaluated by the expressions of PCNA and CyD1. In the 30PLT+MSCs group, the expression of PCNA started to increase at 6 h in both periportal and mid-zonal regions of the liver lobule. Cell proliferation continued to increase up to 72 h, and proliferating cells were predominantly hepatocytes. PCNA positive cells in the hepatic graft constituted approximately 40.3±5.7% in the 30PLT+MSCs group at 72 h after transplantation. By contrast, only a 1.5±0.2% positive rate were detectable in the 30PLT+PBS group (Fig. 6). Similarly, CyD1 positive cells in the hepatic graft comprised approximately 43.2±5.5% in the 30PLT+MSCs group at 72 h after transplantation. But only 1.6±0.4% of intrahepatic cells were CyD1 positive in the 30PLT+PBS group (Fig. 6). These results suggested that 30PLT could obviously suppress the hepatocyte proliferation in liver grafts. However, MSCs therapy revealed a great benefit of promoting the hepatic proliferative responses.

### MSCs therapy increases the activity of transcription factors AP-1 and NF-κB, as well as the expressions of p-c-Jun and CyD1

**Figure 7:**
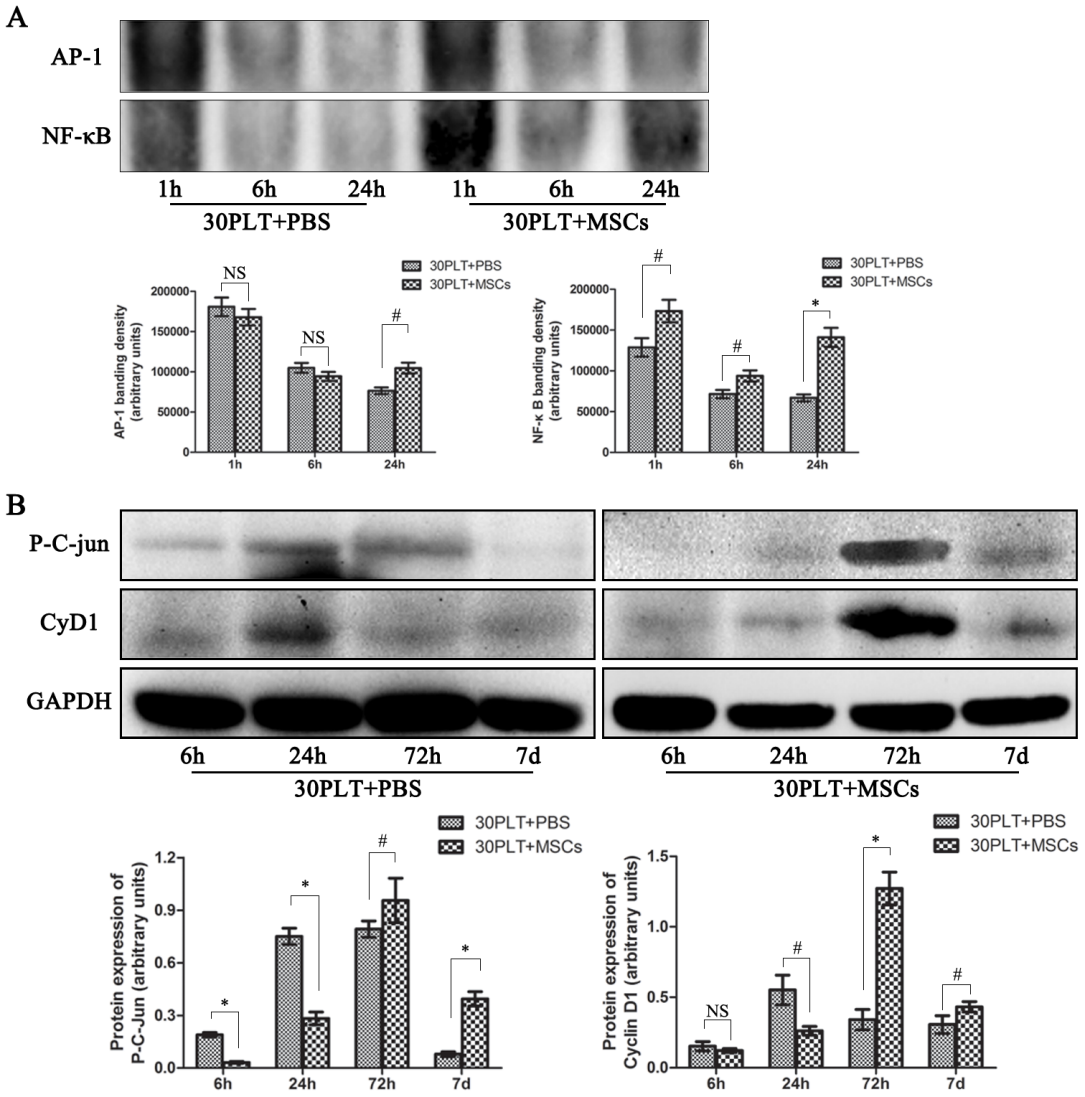
MSCs therapy kept the activity of transcription factors AP-1 and NF-κB, and increased the expressions of phosphorylated c-Jun (p-c-Jun) and Cyclin D1 (CyD1). A. Activation of NF-κB and AP-1 was evaluated by electrophoretic mobility shift assay at 1, 6 and 24 h after reperfusion. Compared with the 30PLT+PBS group, the activity of NF-κB and AP-1 was considerably enhanced at 24 h after reperfusion in the 30PLT+MSCs group; B. Expressions of p-c-Jun and CyD1 were detected by western blotting at 6 h, 24 h, 72 h, and 7 days after reperfusion. Expressions of p-c-Jun and CyD1 were remarkably increased at 72 h after reperfusion. (Mean ± SD; ^#^p<0.05; *p<0.01; NS- nonsignificant.)

The promoter of CyD1 gene contains an AP-1 site. Therefore EMSA assay was used to assess the formation of AP-1/DNA complexes. As is shown, although the AP-1/DNA complexes in both groups were nearly similar at 1 and 6 h after reperfusion, it showed a significant increase after 24 h in the MSCs therapy group (Fig 7A). On the other hand, activation of NF-κB was identified at 1 h after reperfusion in both groups. However its activity in the 30PLT+MSCs group was 1.4-fold of that in the 30PLT+PBS group. Furthermore, it maintained high activity after 24 h, when activity of NF-κB in the 30PLT+MSCs group was 2.1-fold of that in the 30PLT+PBS group (Fig. 7A).

CyD1, an important cell cycle regulator, represented the proliferative response after partial liver transplantation [34]. We also measured the expression of CyD1 at different time points within 7days after SFSLT. As is shown, CyD1 was detectable in liver biopsies within 24 h after reperfusion. A remarkable increase in expression of CyD1 was identified at 72 h after liver transplantation in the 30PLT+MSCs group. However, its expression in the 30PLT+PBS group was considerably suppressed at 72 h, and decreased to 26.8% of that in the 30PLT+MSCs group. Furthermore, CyD1 is an important target of the JNK/c-Jun pathway in driving regeneration [34], [35]. Therefore, the phosphorylation of the JNK target c-Jun was surveyed within 7 days after reperfusion in both groups. Although the peak expression of p-c-Jun was detectable at 72 h after reperfusion in both groups, MSCs therapy increased its expression to 1.43-fold of that in the 30PLT+PBS group (Fig. 7B). Even on 7th day, both the CyD1 and p-c-Jun expressions were still significantly higher in MSCs therapy group than those in the controls. These consistent results indicated MSCs therapy could effectively prolong the intrahepatic proliferative responses to promote liver regeneration.

### MSCs therapy prolongs survival of 30PLT rats

**Figure 8:**
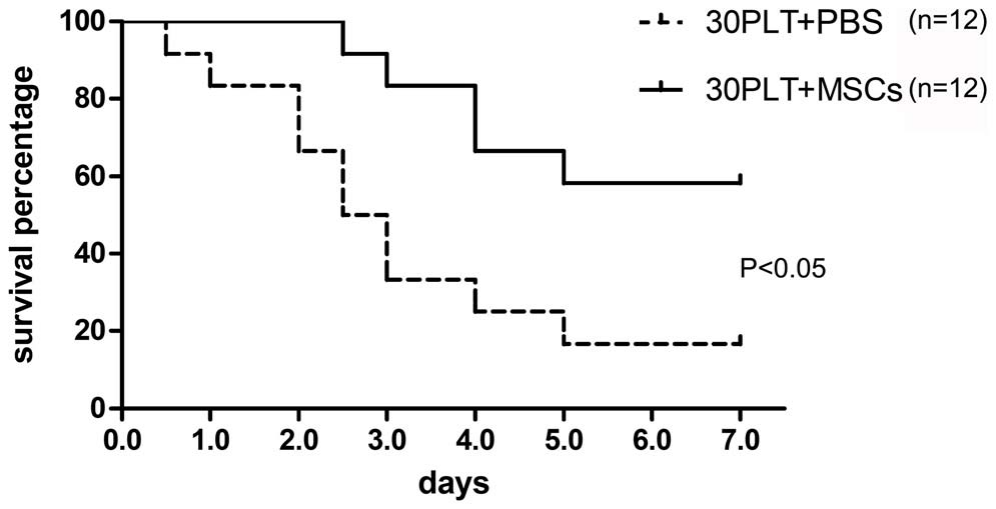
MSCs therapy improved survival rate of 30PLT. 7-day survival rate was 58.3% in the 30PLT+MSCs group. By contrast, it was only 16.7% in the 30PLT+PBS group.

The 7-day survival rates in 30PLT+PBS and 30PLT+MSCs groups were 16.7% (2/12) and 58.3% (7/12) respectively, with a significant difference between the two groups (P<0.05), suggesting that MSCs therapy could evidently improve survival rate of SFSLT (Fig 8).

## Discussion

As one of the most promising therapies for various end-stage hepatic diseases, SFSLT has been widely carried out in clinical practice [1]. However, transient portal hypertension, inevitable hepatic ischemia reperfusion injury and violent inflammation significantly increased postoperative morbidity and mortality [25], [36]. Evidences showed that MSCs therapy was likely to reduce inflammation and promote liver regeneration during acute and chronic liver injuries [7], [12]. Therefore, we hypothesized that MSCs therapy could also effectively resist ischemia reperfusion injury and acute inflammation in SFSLT to promote hepatocytes regeneration, as well as to prolong survival.

Liver has a remarkable regenerative capacity in response to acute injury, and even mature hepatocytes can reenter the cell cycle and undergo several cell divisions to restore the hepatic mass. 70–90% hepatectomy of rats had no effect on life expectancy, suggesting that 10–30% liver tissue could compensate for reduced hepatic function by undergoing cellular regeneration [37]. However, cold ischemia time and anhepatic phase would be inevitable courses in partial liver transplantation. Then excessive blood inflow generates more reactive oxygen species in SFS grafts, increasing the susceptibility of liver cells to apoptotic stimuli and to the mechanical injury associated with transient portal hypertension. Therefore, ischemia reperfusion injury could delay liver regeneration and worsen liver injury in SFSLT, even resulting in small-for-size graft failure [4].

So far, many reports have described cell transplantation as the preferred method of MSC therapy [20], [36]–[38]. Differentiation of MSCs into hepatocyte-like cells has been confirmed in both vitro and vivo [38], [39]. Unfortunately, few engrafted progenitor cells were found in the recipient liver that were able to differentiate into albumin+ cells in liver injury models or reduced size liver transplantation [25], [38], [40]. Moreover, MSCs engraftment and differentiation into hepatocytes within the injured liver did not occur for at least 7 days after infusion, and most recipients died 2–4 days after SFSLT [37], [41]. Such phenomena were also observed in our study, we could only occasionally find a few GFP-positive MSCs on individual slices within 7 days after systematic infusion. Studies conducted by Stock P and Aurich I also consistently described that differentiated human MSCs, which engrafted in the host liver parenchyma of immunocompromised mice after orthotopic transplantation and partial hepatectomy respectively, could be detected at least 2–3 months after cells transplantation [27], [38]. Therefore, MSCs might exert an effect in the early phase of liver injury or SFSLT mainly through paracrine or endocrine mechanisms, as supported by our previous study [25].

Noticeably, direct effects of MSCs in anti-ischemia reperfusion injury and supporting liver regeneration have been demonstrated in several studies [13], [14], [16], [23]. It is well known that hepatic sinusoidal cells play a critical role in the maintenance of hepatocyte function, because sinusoids are the principal vessels involved in the transvascular exchange between blood and parenchymal cells [4]. The vacuolar degeneration and severe swelling of mitochondria in hepatocytes were due to ischemia secondary to the sinusoidal injury, and mitochondria edema would lead to a decreased supply of ATP, which not only serves as an energy supply for regeneration but also affects signal transduction [5]. In this study, MSCs therapy showed a great improvement of these intrahepatic pathologic changes. Both the hepatocytes and sinusoidal lining cells in 30PLT+MSCs grafts basically sustained normal structure with only a slight injury at different time points within 24 h after liver transplantation. This result was consistent with the research conducted by Yu Y [37].

Report by Zhong Z described that insufficiency of energy supply, nutritional factors, proregenerative HGF, TNF-α, and IL-6 formation inhibited liver regeneration in quarter-size grafts [5]. Whereas MSCs treatment could produce a series of cytokines and signal molecules, such as HGF, EGF, IL-6, TNF-α, IL-10, IL-1 receptor antagonists and so on, relevant for cell proliferation, angiogenesis, and anti-inflammatory responses [16], [39]. In our experiment, we confirmed that MSCs infusion notably up-regulated the expressions of genes HGF, Bcl-2, Bcl-XL, IL-10, IP-10, CXCR2, and IL-6, however, down-regulated TNF-α expression. The increased expression of HGF in MSCs+30PLT rats might stimulate liver regeneration and exert cytoprotective effect, which was also demonstrated by the study of Yu Y [37]. As for anti-apoptosis genes Bcl-2 and Bcl-XL, MSCs therapy increased their expressions to resist apoptosis, which would strengthen hepatocyte resistance to ischemia reperfusion injury.

IL-10 was proved to rescue the SFS liver grafts by its anti-inflammatory properties, through inhibition of allograft inflammatory factor-1 (AIF-1) mediated pro-inflammatory and pro-apoptotic activities of the macrophages during the early period after ischemia reperfusion [42]. In this study, enhanced transcription of IL-10 in MSCs therapy rats might limit the acute inflammatory responses induced by ischemia reperfusion. Moreover, IP-10 had a hepatoregenerative effect in the murine model of acute liver injury through the induction of CXCR2 on hepatocytes [33]. Similarly, we found a consistent and sustained up-regulation of both IP-10 and CXCR2 genes in MSCs therapy rats, indicating elevated expression of IP-10 might display its hepatoregenerative effect by stimulating a CXCR2-dependent proliferative response in MSCs therapy group.

It has been widely acknowledged that TNF-α, and IL-6 contribute to activation of transcription factors and provide early signals promoting regeneration [5]. As reported by Schwabe RF and Tiberio L, TNF-α-mediated activation of NF-κB was a key event in liver regeneration [43], and IL-6 stimulated acute-phase synthesis and accelerated activation of the transcription factor Stat 3, which probably played a crucial role in the potentiation of the different protective pathways activated in ischemic-reperfused liver [44]. IL-6 was also directly involved in the generation of antiapoptotic signals through the induction of bcl-2 related protein family members such as Bcl-2, Bcl-xL and Mcl-1 [44]. Thus, the increased transcription of IL-6 in 30PLT+MSCs group might contribute to the high expression of Bcl-2 and Bcl-xL. However, as a pro-inflammatory cytokine, TNF-α could also cause cell injury and apoptosis [43]. Actually, MSCs infusion markedly down-regulated TNF-α expression but up-regulated IL-6 expression at the early phase after reperfusion in our experiment, implying that MSCs therapy probably promoted liver regeneration by limiting the acute inflammation induced by reperfusion. As for the down-regulation of TNF-α, it was also possible that increased transcription of IL-10 in MSCs therapy rats might block AIF-1-mediated pro-inflammatory and pro-apoptotic activities of the macrophages to inhibit the expression of TNF-α [42].

Activation of transcription factors, such as NF-κB, AP-1, STAT3, and expressions of immediate early genes prime quiescent hepatocytes to enter the cell cycle in the priming phase of liver regeneration [5], [45]. In present study, we found that MSCs therapy could sustain comparatively high level of NF-κB and AP-1 at 24 h after reperfusion, and such maintenance would be pivotal to liver graft regeneration in partial liver transplantation [4].

CyD1, as the most prominently up-regulated type D cyclin after partial hepatectomy, responded to mitogenic stimuli and played a critical role in driving cells through the G1 restriction point [34]. It was important that JNK phosphorylated the N-terminal domain of c-Jun, increased its transactivation, thereby up-regulated AP-1-dependent transcription, and then drove CyD1 expression and proliferation during liver regeneration [5], [34]. Research conducted by Zhong Z reported that interruption of JNK/c-Jun and CyD1 signaling would be involved in inhibition of liver regeneration in SFSLT [5]. According to our study, MSCs infusion not only increased the expressions of CyD1 and phosphorylated c-Jun, but also enhanced the activation of AP-1 and NF-κB. These findings indicate that MSCs therapy promoting liver regeneration in small grafts, at least in part, involved the sustained activation of AP-1 and NF-κB, as well as the JNK/c-Jun and CyD1 pathways.

In conclusion, although MSCs were successfully induced into functional hepatocyte-like cells in vitro, their engraftment and differentiation in hepatic grafts was rarely detected within 7 days after SFSLT. However, MSCs infusion could not only alleviate ischemia reperfusion injury and acute inflammation to promote liver regeneration at early stage in rat SFSLT, but also profoundly improved one week survival rate. Based on these findings, we consider that a paracrine effect of cytokines and growth factors secreted by MSCs, which sustained the activation of early proliferative response pathways of JNK/c-Jun, CyD1, and NF-κB, would be the major mechanism for resisting ischemia reperfusion injury and promoting liver regeneration at early stage in SFSLT rat.

## Supporting Information

Checklist S1 (PDF)
